# Supplementary material for: Exploring the limits of pre-trained embeddings in machine-guided protein design: a case study on predicting AAV vector viability
Source: Sci Rep. 2026 Mar 26;16:10974. doi: 10.1038/s41598-026-45458-5 (PMC13039942; doi:10.1038/s41598-026-45458-5)
Supplement: Supplementary file 1 — Supplementary Material 1 [file 41598_2026_45458_MOESM1_ESM.docx]

Rodrigues_Ferraz_*et al.*

**Supplementary Material**

**Supplementary Table 1 – Statistics of the dataset used in this work**

| **Sequence design strategy** | | **Mutations per sequence** | | **Nr of sequences** | | **Viability** |
| --- | --- | --- | --- | --- | --- | --- |
|  |  | *min* | *max* |  |  |  |
| Non-ML-designed | designed | 2 | 42 | 56372 | 92090 | 45% |
|  | single | 1 | 1 | 1112 |  |  |
|  | rand | 2 | 10 | 9885 |  |  |
|  | random_doubles | 2 | 2 | 24721 |  |  |
| ML-designed | cnn_designed_plus_rand_train_seed | 5 | 25 | 1898 | 201426 | 55% |
|  | cnn_designed_plus_rand_train_walked | 5 | 29 | 20759 |  |  |
|  | cnn_rand_doubles_plus_single_seed | 5 | 25 | 2022 |  |  |
|  | cnn_rand_doubles_plus_single_walked | 5 | 29 | 20454 |  |  |
|  | cnn_standard_seed | 5 | 25 | 1924 |  |  |
|  | cnn_standard_walked | 5 | 29 | 20395 |  |  |
|  | lr_designed_plus_rand_train_seed | 5 | 25 | 2030 |  |  |
|  | lr_designed_plus_rand_train_walked | 5 | 29 | 19680 |  |  |
|  | lr_rand_doubles_plus_single_seed | 5 | 25 | 2071 |  |  |
|  | lr_rand_doubles_plus_single_walked | 5 | 29 | 19999 |  |  |
|  | lr_standard_seed | 5 | 25 | 1989 |  |  |
|  | lr_standard_walked | 5 | 29 | 20456 |  |  |
|  | rnn_designed_plus_rand_train_seed | 5 | 25 | 2065 |  |  |
|  | rnn_designed_plus_rand_train_walked | 5 | 29 | 20731 |  |  |
|  | rnn_rand_doubles_plus_singles_seed | 5 | 25 | 2045 |  |  |
|  | rnn_rand_doubles_plus_singles_walked | 5 | 29 | 20154 |  |  |
|  | rnn_standard_seed | 5 | 25 | 1916 |  |  |
|  | rnn_standard_walked | 5 | 29 | 20838 |  |  |

Rodrigues_Ferraz_*et al.*

**Supplementary Material**


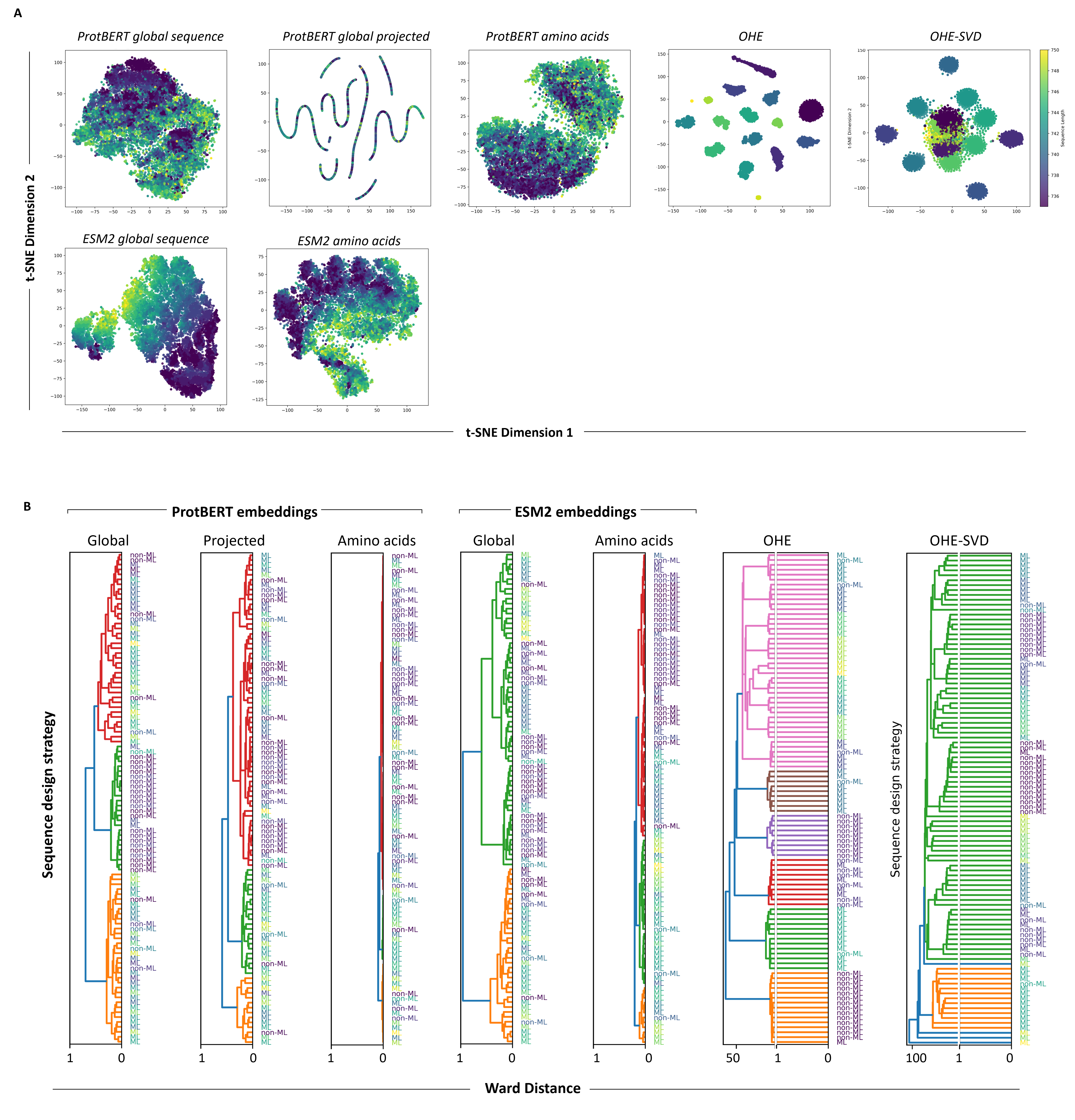


**Supplementary Figure 1.** t-SNE and hierarchical clustering plots of the different representation formats, colored by sequence size of the different representation formats and annotated by design strategy (ML- vs. non-ML-designed sequences). In hierarchical clustering plots note the scale differences between OHE / OHE-SVD and embedding-based representations.

Rodrigues_Ferraz_*et al.*

**Supplementary Material**


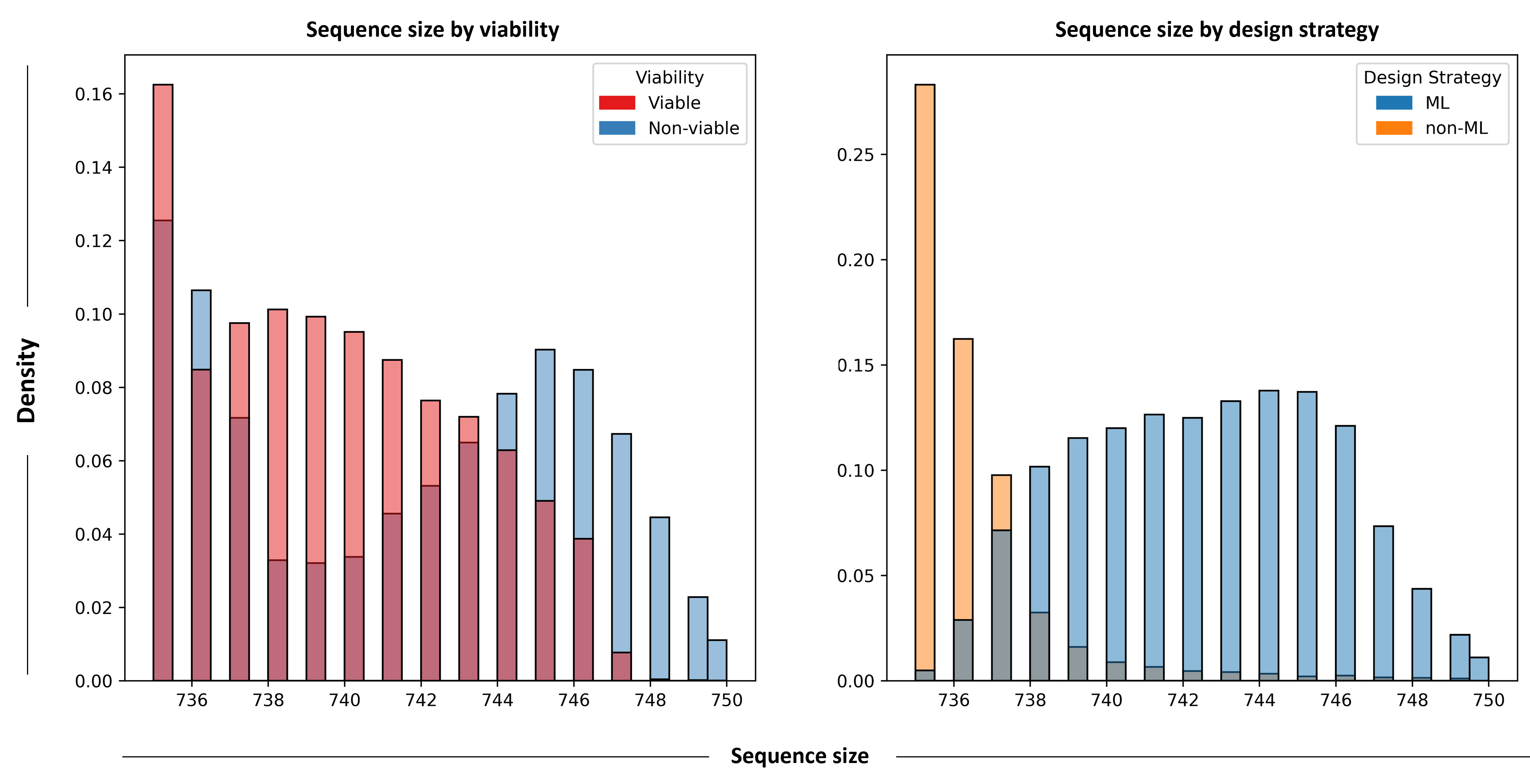


**Supplementary Figure 2.** Histogram of size distributions colored by viability (left panel) or by design strategy (right panel).

Rodrigues_Ferraz_*et al.*

**Supplementary Material**

**Supplementary Table 2 – Test metrics for the different classifier models using ProtBERT embeddings**

| **ML model** | **Representation format** | **Accuracy** | | **Precision** | | **Recall** | | **F1 Score** | |
| --- | --- | --- | --- | --- | --- | --- | --- | --- | --- |
|  |  | **Mean** | **StDev** | **Mean** | **StDev** | **Mean** | **StDev** | **Mean** | **StDev** |
| **Random forests** | Global sequence embedding | 0.865 | 0.002 | 0.865 | 0.002 | 0.876 | 0.002 | 0.870 | 0.002 |
|  | Projected embedding | 0.569 | 0.002 | 0.553 | 0.002 | 0.872 | 0.012 | 0.677 | 0.002 |
|  | Amino acids embedding | 0.867 | 0.002 | 0.866 | 0.002 | 0.878 | 0.002 | 0.872 | 0.001 |
|  | Global sequence embedding (SVD) | 0.846 | 0.001 | 0.847 | 0.002 | 0.857 | 0.002 | 0.852 | 0.001 |
|  | Projected embedding (SVD) | 0.598 | 0.006 | 0.578 | 0.006 | 0.827 | 0.022 | 0.681 | 0.006 |
|  | Amino acids embedding (SVD) | 0.860 | 0.002 | 0.860 | 0.002 | 0.872 | 0.002 | 0.866 | 0.002 |
|  | Global + amino acids embedding (SVD) | 0.855 | 0.002 | 0.854 | 0.002 | 0.868 | 0.002 | 0.861 | 0.002 |
|  | OHE (SVD) | 0.923 | 0.001 | 0.908 | 0.001 | 0.947 | 0.001 | 0.927 | 0.001 |
| **Logistic regression** | Global sequence embedding | 0.854 | 0.002 | 0.849 | 0.002 | 0.873 | 0.002 | 0.861 | 0.001 |
|  | Projected embedding | 0.518 | 0.000 | 0.518 | 0.000 | 1.000 | 0.000 | 0.682 | 0.000 |
|  | Amino acids embedding | 0.851 | 0.001 | 0.843 | 0.002 | 0.876 | 0.002 | 0.859 | 0.001 |
|  | Global sequence embedding (SVD) | 0.913 | 0.001 | 0.909 | 0.002 | 0.926 | 0.001 | 0.917 | 0.001 |
|  | Projected embedding (SVD) | 0.615 | 0.002 | 0.619 | 0.002 | 0.670 | 0.004 | 0.643 | 0.003 |
|  | Amino acids embedding (SVD) | 0.922 | 0.001 | 0.918 | 0.001 | 0.933 | 0.001 | 0.925 | 0.001 |
|  | Global + amino acids embedding (SVD) | 0.922 | 0.001 | 0.918 | 0.001 | 0.933 | 0.001 | 0.926 | 0.001 |
|  | OHE (SVD) | 0.930 | 0.001 | 0.917 | 0.001 | 0.950 | 0.001 | 0.933 | 0.001 |
| **Multilayer perceptron** | Global embedding | 0.871 | 0.021 | 0.872 | 0.054 | 0.889 | 0.074 | 0.876 | 0.025 |
|  | Projected embedding | 0.513 | 0.012 | 0.449 | 0.179 | 0.867 | 0.346 | 0.591 | 0.236 |
|  | Amino acids embedding | 0.897 | 0.013 | 0.896 | 0.044 | 0.912 | 0.056 | 0.901 | 0.014 |
|  | Global embedding (SVD) | 0.933 | 0.001 | 0.934 | 0.002 | 0.937 | 0.002 | 0.936 | 0.001 |
|  | Projected embedding (SVD) | 0.610 | 0.006 | 0.610 | 0.004 | 0.687 | 0.031 | 0.646 | 0.013 |
|  | Amino acids embedding (SVD) | 0.938 | 0.001 | 0.938 | 0.002 | 0.942 | 0.003 | 0.940 | 0.001 |
|  | Global + amino acids embedding (SVD) | 0.938 | 0.001 | 0.938 | 0.003 | 0.942 | 0.004 | 0.940 | 0.001 |
|  | OHE (SVD) | 0.946 | 0.001 | 0.947 | 0.005 | 0.948 | 0.006 | 0.947 | 0.001 |

**Supplementary Table 3 – Test metrics for the different classifier models using ESM2 embeddings**

| **ML model** | **Representation format** | **Accuracy** | | **Precision** | | **Recall** | | **F1 Score** | |
| --- | --- | --- | --- | --- | --- | --- | --- | --- | --- |
|  |  | **Mean** | **StDev** | **Mean** | **StDev** | **Mean** | **StDev** | **Mean** | **StDev** |
| **Random forests** | Global sequence embedding | 0.881 | 0.001 | 0.876 | 0.002 | 0.898 | 0.002 | 0.887 | 0.001 |
|  | Amino acids embedding | 0.875 | 0.001 | 0.871 | 0.002 | 0.891 | 0.002 | 0.881 | 0.001 |
|  | Global sequence embedding (SVD) | 0.877 | 0.001 | 0.873 | 0.002 | 0.893 | 0.002 | 0.883 | 0.001 |
|  | Amino acids embedding (SVD) | 0.869 | 0.002 | 0.866 | 0.003 | 0.883 | 0.002 | 0.874 | 0.002 |
|  | Global + amino acids embedding (SVD) | 0.872 | 0.002 | 0.868 | 0.003 | 0.888 | 0.002 | 0.878 | 0.002 |
|  | OHE (SVD) | 0.922 | 0.001 | 0.907 | 0.002 | 0.947 | 0.001 | 0.927 | 0.001 |
| **Logistic regression** | Global sequence embedding | 0.805 | 0.002 | 0.802 | 0.003 | 0.828 | 0.002 | 0.815 | 0.002 |
|  | Amino acids embedding | 0.813 | 0.004 | 0.806 | 0.004 | 0.842 | 0.004 | 0.824 | 0.003 |
|  | Global sequence embedding (SVD) | 0.938 | 0.001 | 0.932 | 0.001 | 0.950 | 0.001 | 0.941 | 0.001 |
|  | Amino acids embedding (SVD) | 0.935 | 0.001 | 0.929 | 0.001 | 0.946 | 0.001 | 0.937 | 0.001 |
|  | Global + amino acids embedding (SVD) | 0.939 | 0.001 | 0.933 | 0.001 | 0.950 | 0.001 | 0.942 | 0.001 |
|  | OHE (SVD) | 0.930 | 0.001 | 0.917 | 0.001 | 0.949 | 0.001 | 0.933 | 0.001 |
| **Multilayer perceptron** | Global embedding | 0.874 | 0.061 | 0.849 | 0.061 | 0.831 | 0.03 | 0.837 | 0.033 |
|  | Amino acids embedding | 0.911 | 0.011 | 0.899 | 0.006 | 0.888 | 0.011 | 0.894 | 0.006 |
|  | Global embedding (SVD) | 0.955 | 0.002 | 0.952 | 0.001 | 0.949 | 0.002 | 0.950 | 0.001 |
|  | Amino acids embedding (SVD) | 0.952 | 0.002 | 0.949 | 0.001 | 0.946 | 0.002 | 0.947 | 0.001 |
|  | Global + amino acids embedding (SVD) | 0.955 | 0.002 | 0.951 | 0.001 | 0.948 | 0.002 | 0.950 | 0.001 |
|  | OHE (SVD) | 0.957 | 0.003 | 0.953 | 0.001 | 0.950 | 0.003 | 0.952 | 0.001 |

Rodrigues_Ferraz_*et al.*

**Supplementary Material**


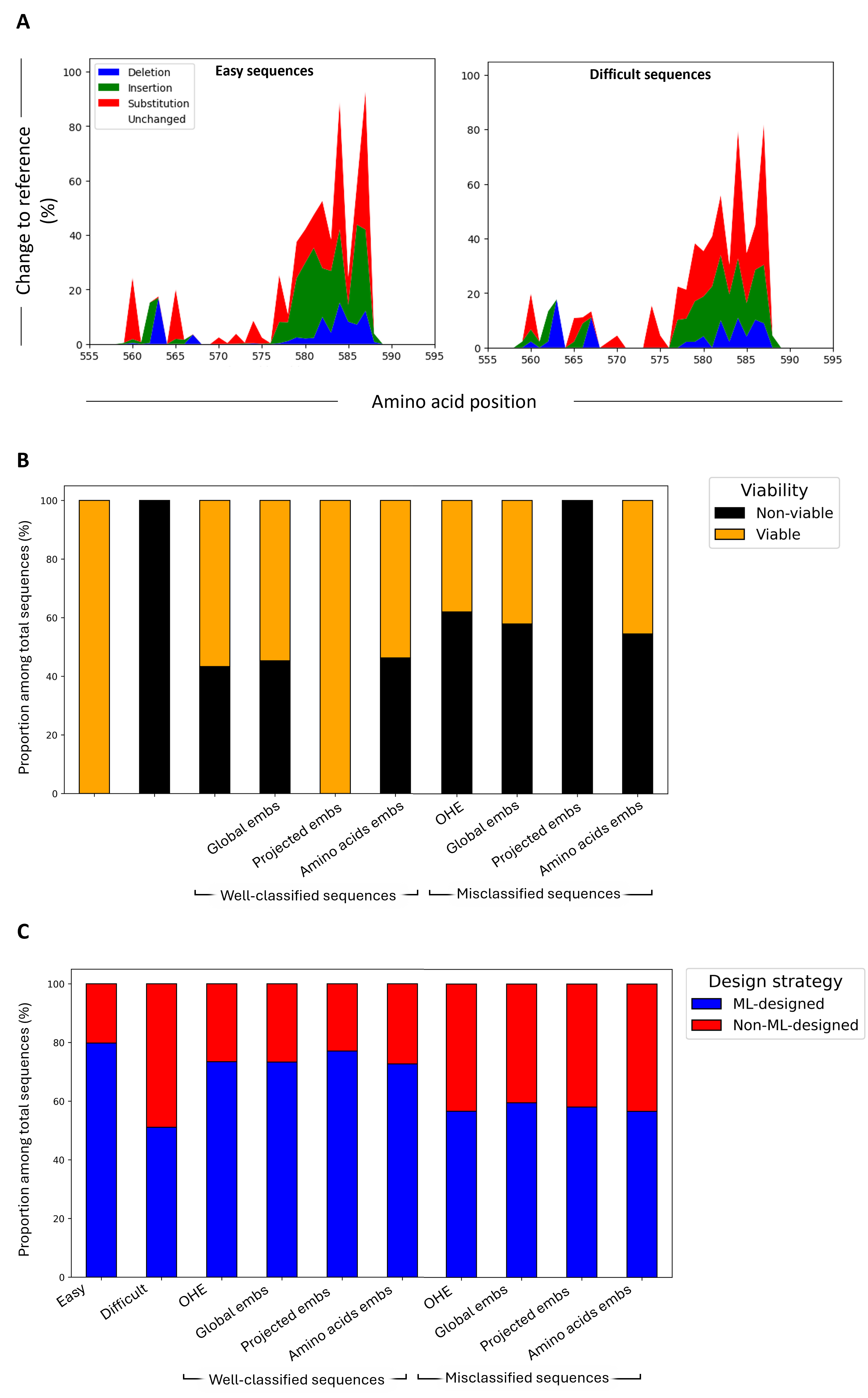


**Suplementary Figure 3.** (A) Mutational landscape analysis of easy vs difficult sequences when using classifier-representation pairs featuring ProtBERT embeddings only. (B, C) Composition of sequence groups when using classifier-representation pairs conatining only ProtBERT embeddings. Groups of sequences sharing functional features (classification difficulty and correctness across all model-representation pairs featuring the specified representation format) are shown in terms of viability (B) and design strategy (C).

Rodrigues_Ferraz_*et al.*

**Supplementary Material**

**
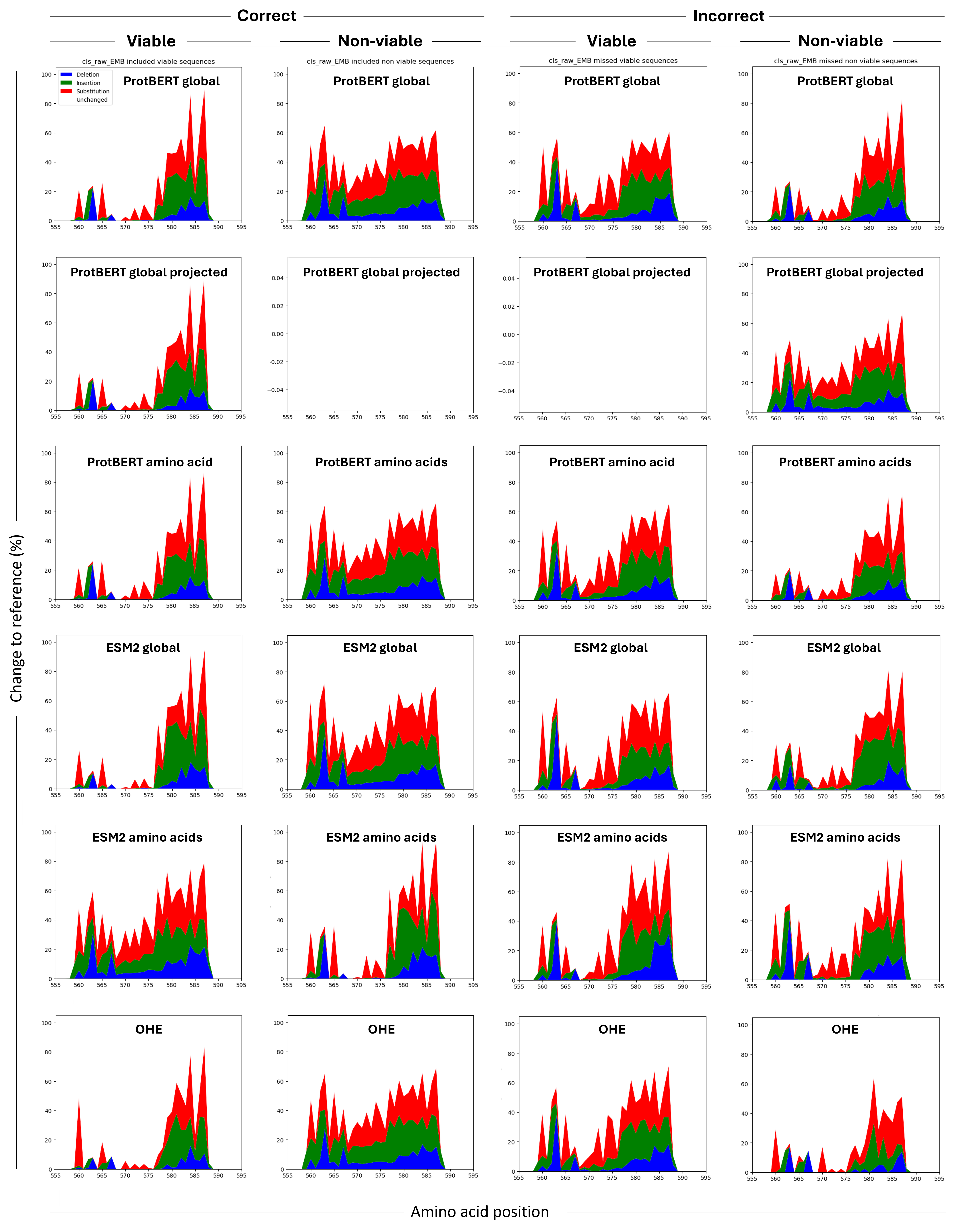
**

**Supplementary Figure 4.** Mutational landscape analysis. Distribution of mutation types (deletions, insertions, and substitutions) across amino acid positions 561 to 588 in the targeted region, expressed as the percentage of change relative to the reference sequence. Data are shown for groups of sequences that were correctly or incorrectly classified across all representation formats of the same type, stratified by viability.

Rodrigues_Ferraz_*et al.*

**Supplementary Material**

**
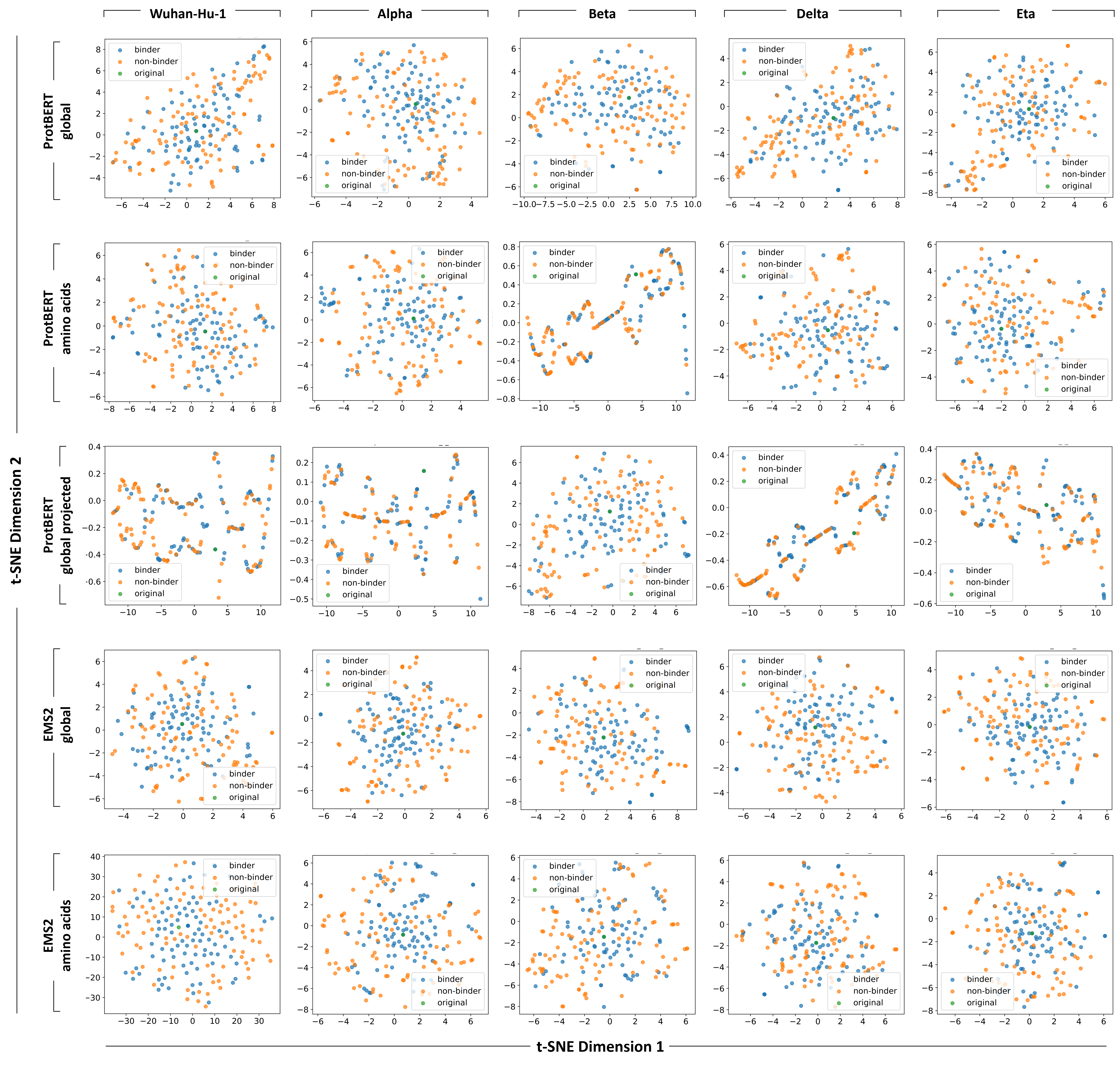
**

**Supplementary Figure 5.** t-SNE visualizations of ProtBERT and ESM2 embeddings for a separate dataset comprising deep mutational scanning variants of the SARS-CoV-2 spike protein across five variant backbones (Wuhan-Hu-1, Alpha, Beta, Delta, and Eta), annotated by binding activity to the target. The dataset was obtained from T. Starr *et al.* (2022) (reference 43 in the main manuscript). Variants were classified as binders or non-binders using a data-driven thresholding approach based on the distribution of measured binding scores. For each variant backbone, binding scores from all measured sequences were modeled as a mixture of two Gaussian distributions representing (i) strong binders (high binding scores) and (ii) weak or non-binders (low binding scores). The intersection point of the two Gaussians was used as the threshold separating binders from non-binders. To account for measurement noise and borderline cases, a ±5% buffer around this threshold was applied. Each plot shows a random sampling of 100 binders, 100 non-binders, and the original (wild-type) sequence for each variant backbone.

Rodrigues_Ferraz_*et al.*

**Supplementary Material**

**
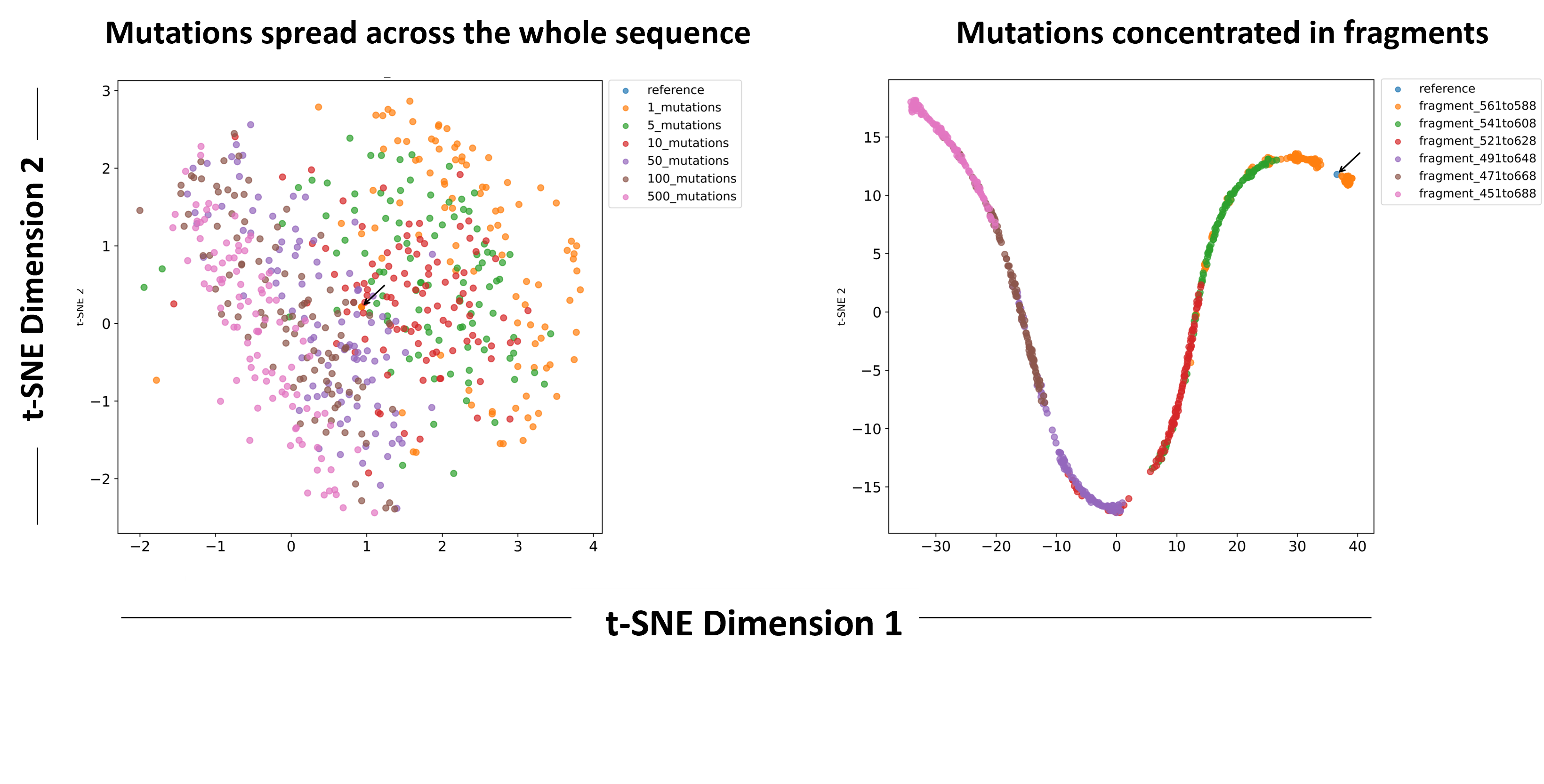
**

**Supplementary Figure 6.** t-SNE plots of 1280-component OHE-SVD for sequences with either dispersed mutations across the entire sequence (left panel) or mutations concentrated in defined target regions (right panel). For dispersed mutations, the indicated number of substitutions was introduced randomly; for targeted fragments, every position within the specified regions was mutated. These plots complement those in Figure 6 of the main manuscript and were omitted there for simplicity, as the results and conclusions are consistent with OHE-SVD using 1024 dimensions. Arrows indicate the reference (unmutated) sequence in all panels.

Rodrigues_Ferraz_*et al.*

**Supplementary Material**

**Supplementary Table 4 – Test metrics for fine-tuned models using ProtBERT embeddings**

| **Representation format** | **Accuracy** | | **Precision** | | **Recall** | | **F1 Score** | |
| --- | --- | --- | --- | --- | --- | --- | --- | --- |
|  | **Mean** | **StDev** | **Mean** | **StDev** | **Mean** | **StDev** | **Mean** | **StDev** |
| Global sequence embedding | 0.955 | 0.002 | 0.958 | 0.013 | 0.955 | 0.012 | 0.957 | 0.002 |
| Projected embedding | 0.952 | 0.002 | 0.948 | 0.012 | 0.959 | 0.013 | 0.954 | 0.002 |
| Amino acids embedding | 0.947 | 0.002 | 0.941 | 0.011 | 0.958 | 0.011 | 0.949 | 0.002 |

**Supplementary Table 5 – Test metrics for fine-tuned models using ESM2embeddings**

| **Representation format** | **Accuracy** | | **Precision** | | **Recall** | | **F1 Score** | |
| --- | --- | --- | --- | --- | --- | --- | --- | --- |
|  | **Mean** | **StDev** | **Mean** | **StDev** | **Mean** | **StDev** | **Mean** | **StDev** |
| Global sequence embedding | 0.951 | 0.002 | 0.952 | 0.015 | 0.955 | 0.014 | 0.953 | 0.002 |
| Amino acids embedding | 0.949 | 0.005 | 0.951 | 0.018 | 0.952 | 0.017 | 0.951 | 0.004 |
